# SPP1‐ITGα5/β1 Accelerates Calcification of Nucleus Pulposus Cells by Inhibiting Mitophagy via Ubiquitin‐Dependent PINK1/PARKIN Pathway Blockade

**DOI:** 10.1002/advs.202411162

**Published:** 2024-12-25

**Authors:** Hanwen Gu, Qi Li, Zhenchuan Liu, Yanlin Li, Kaiwen Liu, Xiangzhen Kong, Yuanqiang Zhang, Qunbo Meng, Kangle Song, Qing Xie, Yuan Gao, Lei Cheng

**Affiliations:** ^1^ Department of Orthopedic Qilu Hospital of Shandong University Jinan Shandong 250012 China; ^2^ Department of Pharmacy Qilu Hospital Cheeloo College of Medicine Shandong University Jinan 250012 China; ^3^ Department of Orthopedic Qilu Hospital of Shandong University Jinan Shandong 250012 China

**Keywords:** calcification, integrin α5β1, intervertebral disc degeneration, mitophagy, nucleus pulposus cells, secreted phosphoprotein 1

## Abstract

Low back pain (LBP) caused by nucleus pulposus degeneration and calcification leads to great economic and social burden worldwide. Unexpectedly, no previous studies have demonstrated the association and the underlying mechanism between nucleus pulposus tissue degeneration and calcification formation. Secreted Phosphoprotein 1 (SPP1) exerts crucial functions in bone matrix mineralization and calcium deposition. Here, a novel function of SPP1 is reported, namely that it can aggravate nucleus pulposus cells (NPs) degeneration by negatively regulating extracellular matrix homeostasis. The degenerated NPs have a higher mineralization potential, which is achieved by SPP1. Mechanistically, SPP1 can accelerate the degeneration of nucleus pulposus cells by activating integrin α5β1 (ITGα5/β1), aggravating mitochondrial damage and inhibiting mitophagy. SPP1‐ITGα5/β1 axis inhibits mitophagy by PINK1/PARKIN pathway blockade. In conclusion, SPP1 activates ITGα5/β1 to inhibit mitophagy, accelerates NPs degeneration, and induces calcification, thereby leading to intervertebral disc degeneration (IVDD) and calcification, identifying the potentially unknown mechanism and relationship between IVDD and calcification. Important insights are provided into the role of SPP1 in nucleus pulposus calcification in IVDD by inducing nucleus pulposus cell senescence through inhibition of mitophagy and may help develop potential new strategies for IVDD treatment.

## Graphical abstract

Secreted Phosphoprotein 1 (SPP1) serves as a novel mitophagy inhibitor in the transformation from degeneration of nucleus pulposus cells to calcification. SPP1 in degenerative NPs mediated intracellular mitochondrial dysfunction and inhibits mitophagy by activating ITGα5/β1, accelerating cellular aging, which in turn affects extracellular matrix homeostasis.

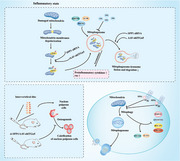

## 1 Introduction

LBP is a leading cause of disability, causing decreased productivity and reduced life expectancy on a global scale, with 40% of the general population suffering.^[^ ^1^ ^]^ IVDD is considered to lead to LBP,^[^ ^2^ ^]^ characterized by extracellular matrix disturbance, nucleus pulposus fibrosis, and dehydration, calcification, etc.^[^ ^3^ ^]^ The propensity for nucleus pulposus calcification is notably heightened in degenerated discs.^[^ ^4^ ^]^ The visualization of Imaging tests, mainly X‐ray imaging, may be partially masked by the presence of vertebrae.^[^ ^5^ ^]^ Therefore, IVD calcification is an overlooked disc phenotype, tending to be aggravated as the grade of disc degeneration increased.^[^ ^4^ ^]^ Currently, the specific mechanism of nucleus pulposus tissue calcification remains unknown. A better grasp of the cellular and molecular mechanisms underlying nucleus pulposus calcification is crucial for devising effective strategies to prevent disease progression.

Mitophagy is a specialized form of autophagy that facilitates the renewal of damaged mitochondria, which is essential for maintaining mitochondrial homeostasis.^[^ ^6^ ^]^ Impaired mitophagy often leads to a series of pathophysiological processes, such as apoptosis, senescence, and cell degeneration.^[^ ^6^, ^7^ ^]^ It was found that the mitophagy receptor BNIP3 was critical for the regulation of metabolic homeostasis and mitochondrial function in the nucleus pulposus cells.^[^ ^8^ ^]^ Mitochondrial dynamics and mitophagy was able to rejuvenate intervertebral disc.^[^ ^9^ ^]^ Therefore, maintenance of mitophagy may be one of the key strategies to rescue IVDD.

SPP1 gene, involved in bone matrix mineralization and extracellular matrix destruction, encodes the OPN protein.^[^ ^10^ ^]^ In a recent study, it was found that SPP1 was crucial for the early development of intervertebral discs in both humans and mice.^[^ ^11^ ^]^ A clinical investigation of degenerated intervertebral discs revealed a more than 400‐fold increase in SPP1 expression.^[^ ^12^ ^]^ A substantial body of single‐cell sequencing data suggested that SPP1 was implicated in the fate regulation of nucleus pulposus progenitor cells/mesenchymal stem cells, macrophages, and nucleus pulposus cells in the intervertebral disc, and played a pro‐inflammatory and immune infiltration role in IVDD, which may further aggravate intervertebral disc degeneration.^[^ ^13^ ^]^ It follows that SPP1 may be involved in the calcification of the nucleus pulposus during the process of intervertebral disc degeneration.

ITG plays a critical role in mediating cell adhesion and signaling, and are essential for a diverse array of biological functions, with numerous subunits.^[^ ^14^ ^]^ It was found that ITGα2/β1 played a significant role in the regulation of ECM remodeling, the senescence of nucleus pulposus cells, and the apoptosis of annulus fibrosus cells.^[^ ^15^ ^]^ A recent single‐cell sequencing analysis demonstrated that the level of SPP1 was upregulated in degenerative nucleus pulposus populations, in which integrin subunits ITGα5/β1 may be potentially associated with this term.^[^ ^16^ ^]^ There is a paucity of research regarding the mechanisms by which the integrin receptor family contributes to intervertebral disc degeneration and calcification.

In this study, we revealed that elevated levels of SPP1 and ITGα5 were observed in nucleus pulposus tissues exhibiting Pfirrmann grade IV degeneration. The mitochondrial membrane potential reduction and mitochondrial autophagy inhibition of rat NPs was caused by SPP1, leading to a decrease in the clearance of intracellular ROS and their accumulation, accelerating the cell aging and degeneration, and subsequently causing extracellular matrix disorder and calcification, in which ITGα5/β1 was also involved. This study elucidated the function and regulatory mechanisms of SPP1‐ITGα5/β1 in IVDD by inhibiting mitophagy and proposed a potential therapeutic approach for strategy for intervertebral disc degeneration and calcification.

## 2 Results

### 2.1 Increased SPP1 Expression in Human Degenerative Intervertebral Discs Synchronized with Calcification and Disturbance of Extracellular Matrix

**Figure 1:**
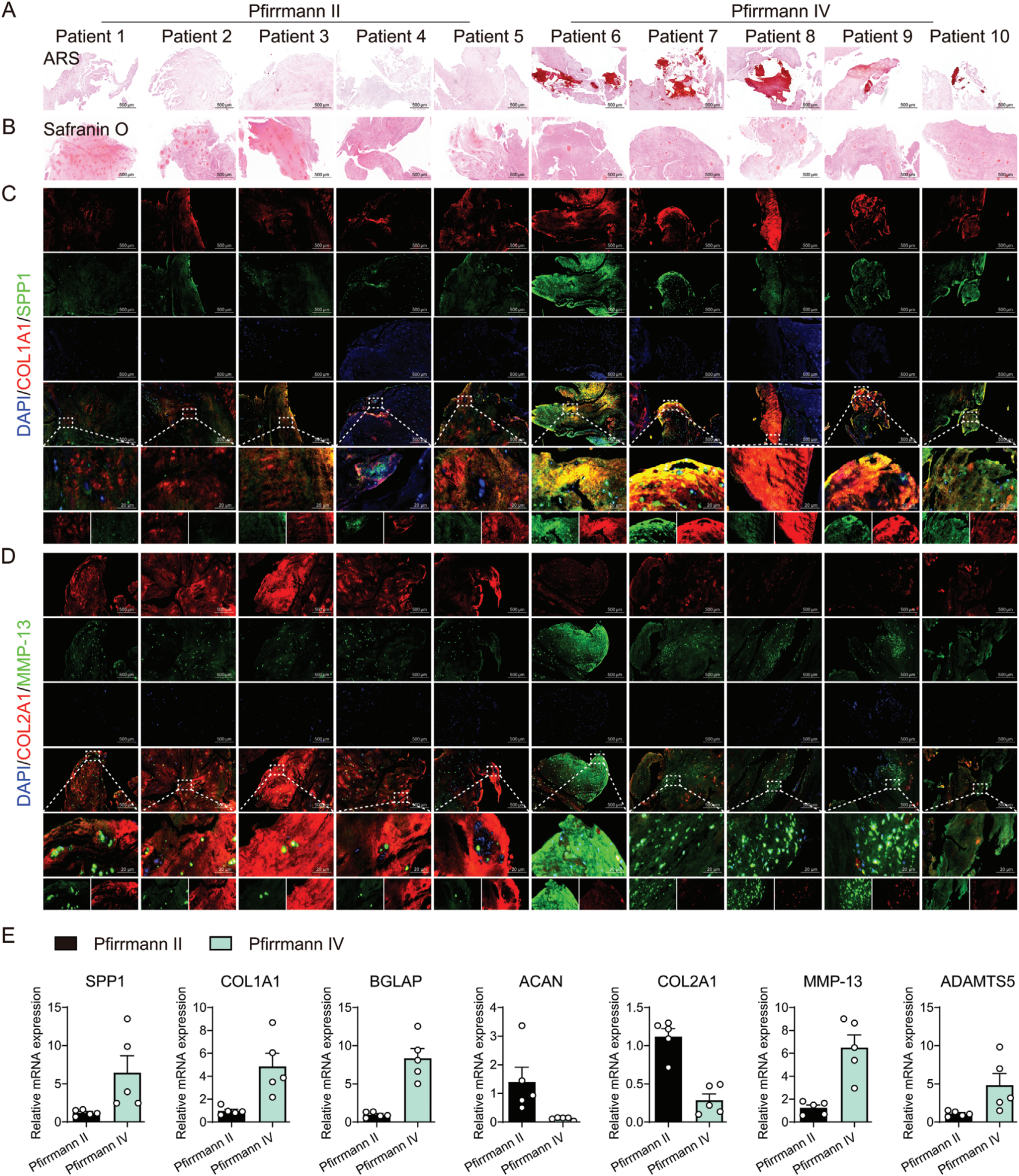
Increased SPP1 expression in degenerative intervertebral discs synchronized with calcification and disturbance of extracellular mechanism. A) ARS staining, 500 µm, *n* = 5; B) Safranin O staining, 500 µm, *n* = 5; C) Representative immunofluorescence images of COL1A1 and SPP1, 500 µm, 20 µm, *n* = 5; D) Representative immunofluorescence images of COL2A1 and MMP13, 500 µm, 20 µm, *n* = 5; E) Relative mRNA level of SPP1, COL1A1, BGLAP, COL2A1, ACAN, MMP13, ADAMTS5, *n* = 5. ^*^ *p* < 0.05, ^**^ *p* < 0.01 versus Pfirrmann II.

Clinically, calcified tissue was often found in the highly degraded nucleus pulposus.^[^ ^17^ ^]^ As a bone matrix protein, SPP1 was closely related to bone formation and development.^[^ ^18^ ^]^ To investigate the role of SPP1 in disc calcification, we collected degenerated disc with Pfirrmann II and Pfirrmann IV for pathological examination. The ARS staining results showed more calcium deposition and calcification in Pfirrmann IV nucleus pulposus, compared with Pfirrmann II (**Figure** **1**A). The results of safranin O staining in Pfirrmann IV nucleus pulposus indicated lighter staining, decreased deposition of proteoglycan and collagen, significant dehydration, and enhanced fibrosis (Figure 1B). Additionally, for Pfirrmann IV nucleus pulposus, the potential osteogenesis and extracellular matrix homeostasis were subsequently confirmed by immunofluorescence, which showed dramatic increases in COL1A1, SPP1, and MMP13 fluorescence intensity, indicative of calcification and extracellular matrix degeneration intensification, and decreased COL2A1 fluorescence intensity, indicative of extracellular matrix synthesis reduction (Figure 1C,D). Expression of key markers of potential osteogenesis (SPP1, BGLAP, and COL1A1) and extracellular matrix degeneration (MMP13 and ADAMTS5) was also elevated in Pfirrmann IV nucleus pulposus, contrary to the decreased expression of extracellular matrix synthesis markers (ACAN and COL2A1), compared with Pfirrmann II (Figure 1E).

### 2.2 Increased SPP1 was Involved in Degenerative Intervertebral Discs of Rats

**Figure 2:**
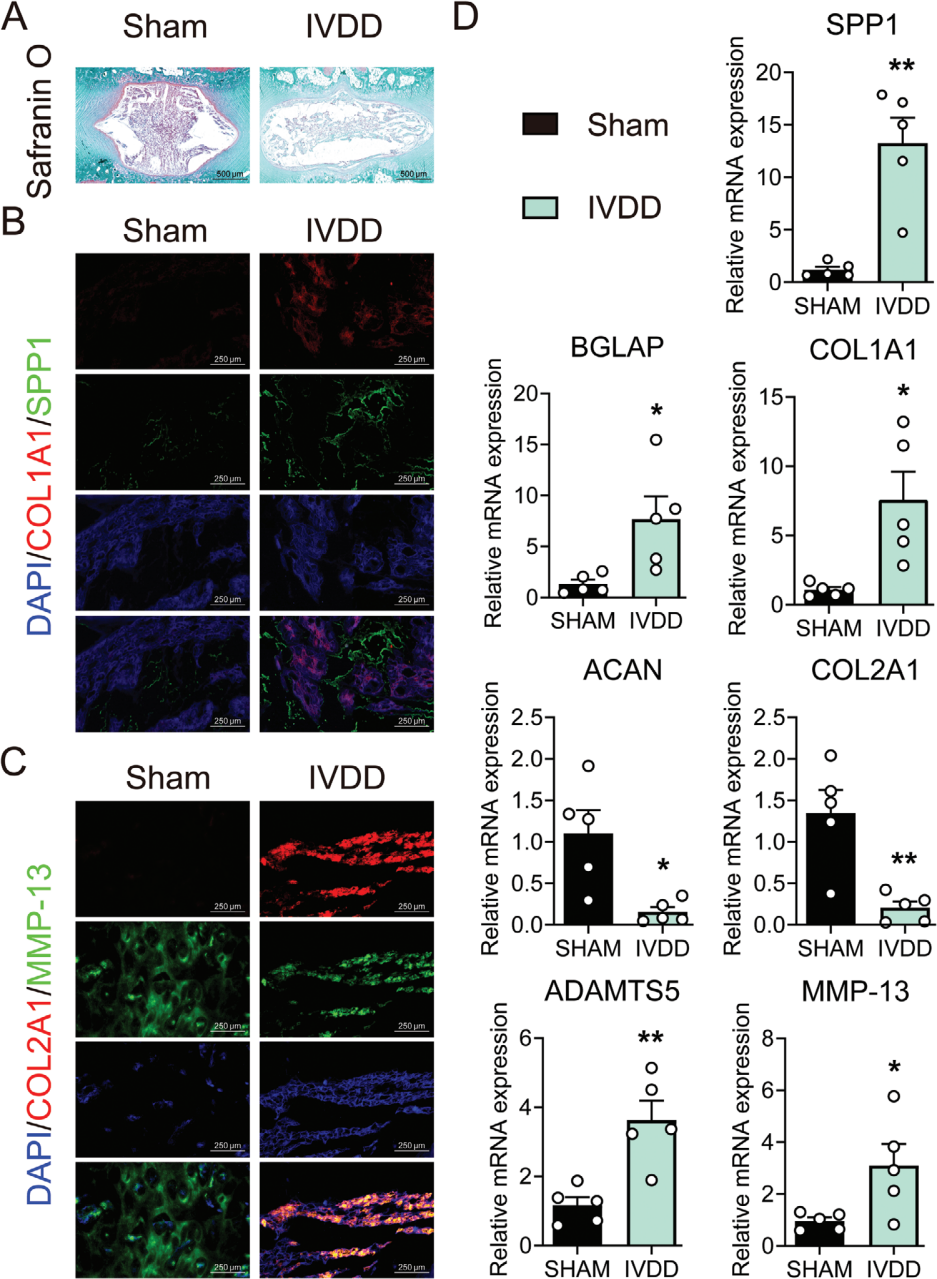
Increased SPP1 was involved in degenerative intervertebral discs of rats. A) Safranin O staining, 500 µm, *n* = 5; B) Representative immunofluorescence images of COL1A1 and SPP1, 250 µm, *n* = 5; C) Representative immunofluorescence images of COL2A1 and MMP13, 250 µm, *n* = 5; D) Relative mRNA level of SPP1, COL1A1, BGLAP, COL2A1, ACAN, MMP13, ADAMTS5, *n* = 5. ^*^ *p* < 0.05, ^**^ *p* < 0.01 versus Sham.

The rat needle model was used to verify the findings above. Compared with sham group rats, the nucleus pulposus of IVDD group rats indicated lighter safranin O staining (**Figure** **2**A), and results of immunofluorescence and RT‐qPCR were human‐like (Figure 2B–D). These results indicated an increased calcification tendency and an imbalance in extracellular matrix synthesis and degradation in degenerative nucleus pulposus, which were associated with high expression of SPP1.

### 2.3 SPP1 Promoted Osteogenic Differentiation of Nucleus Pulposus Cells and Disrupted Extracellular Matrix Homeostasis Under Inflammatory Conditions

**Figure 3:**
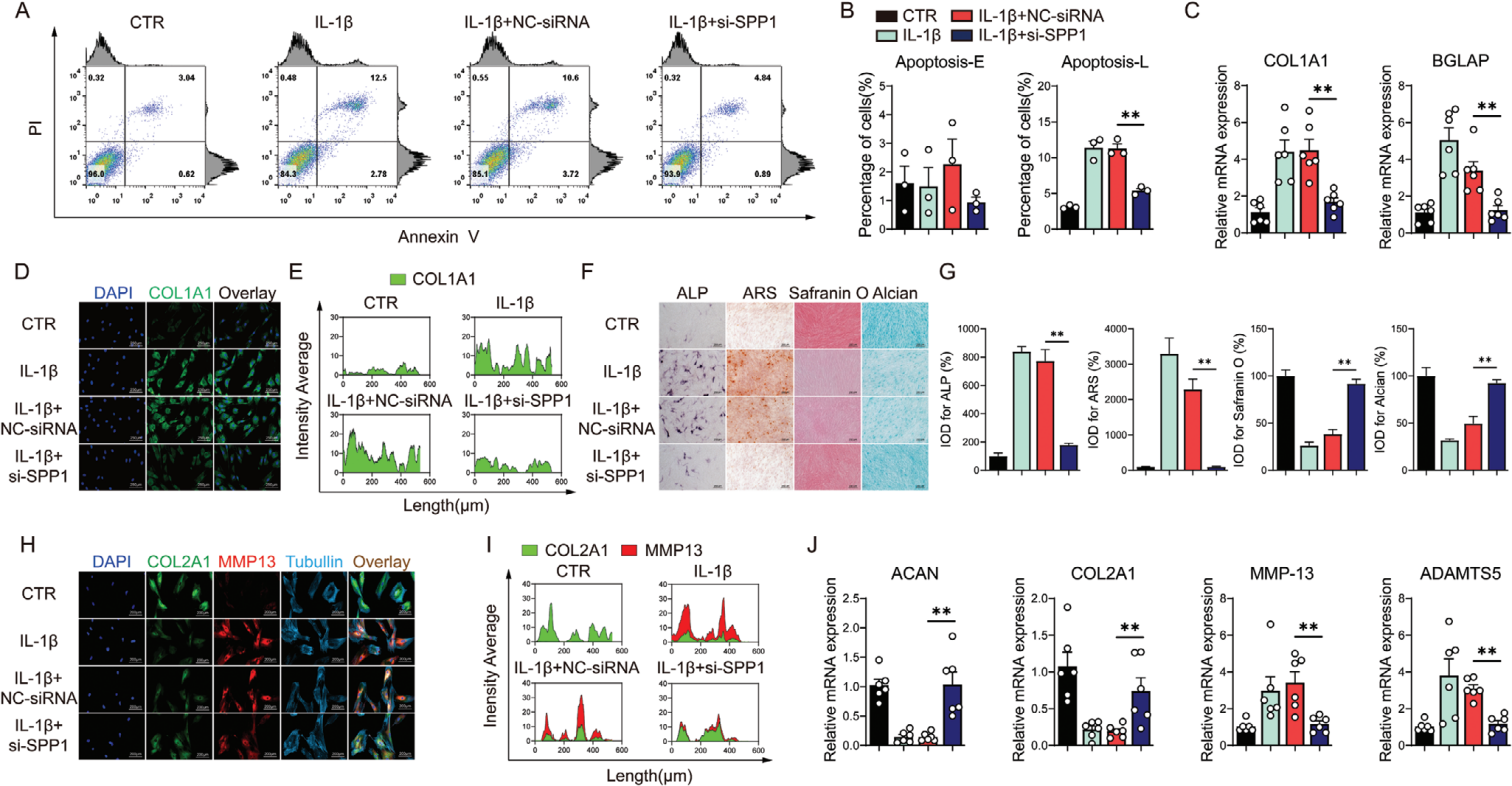
SPP1 promoted osteogenic differentiation of nucleus pulposus cells and disrupted extracellular matrix homeostasis under inflammatory conditions. A,B) Apoptosis flow cytometry, *n* = 3; C) Relative mRNA level of COL1A1 and BGLAP, *n* = 6; D,E) Representative immunofluorescence images of COL1A1, 250 µm, *n* = 3; F,G) ALP staining, ARS staining, Safranin O staining and alcian staining, 200×, *n* = 3; H,I) Representative immunofluorescence images of COL2A1 and MMP13, 200 µm, *n* = 3; J) Relative mRNA level of ACAN, COL2A1, MMP13 and ADAMTS5, *n* = 6. ^*^ *p* < 0.05, ^**^ *p* < 0.01 versus IL‐1β+NC‐siRNA.

SPP1 has a potential association with calcification of nucleus pulposus in intervertebral discs, and IL‐1β‐induced inflammatory NPs model was used for experiments in vitro, and the efficiency of SPP1 knockdown was verified (Figure S1, Supporting Information). The results of flow cytometry suggested that siRNA‐mediated knockdown of SPP1 in NPs significantly impaired apoptosis caused by IL‐1β, especially in the late (end‐stage) apoptotic cells (Annexin V–FITC^+^/PI^+^) (**Figure** **3**A,B). Upon osteogenic induction, COL1A1 and BGLAP mRNA expression (Figure 3C) and COL1A1 protein expression (Figure 3D,E) significantly decreased upon SPP1 knockdown. The ALP staining (3 days induction) and ARS staining (10 days induction) revealed that knockdown of SPP1 exhibited substantial osteogenesis decreases of NPs under inflammatory conditions (Figure 3F,G). Further, safranin O staining and alcian staining were conducted to confirm that degeneration of NPs has been alleviated, manifested as SPP1 knockdown enhanced staining in the inflammatory state, indicating increased glycosaminoglycan and mucopolysaccharide synthesis (Figure 3F,G). ACAN, COL2A1, MMP13, and ADAMTS5 mRNA expression and COL2A1 protein expression revealed an increase of extracellular matrix synthesis and a decrease of degradation after SPP1 knockdown in degenerated NPs (Figure 3H–J).

### 2.4 SPP1 Induced Senescence Through ROS Accumulation Mediated by Mitophagy Reduction in NPs

**Figure 4:**
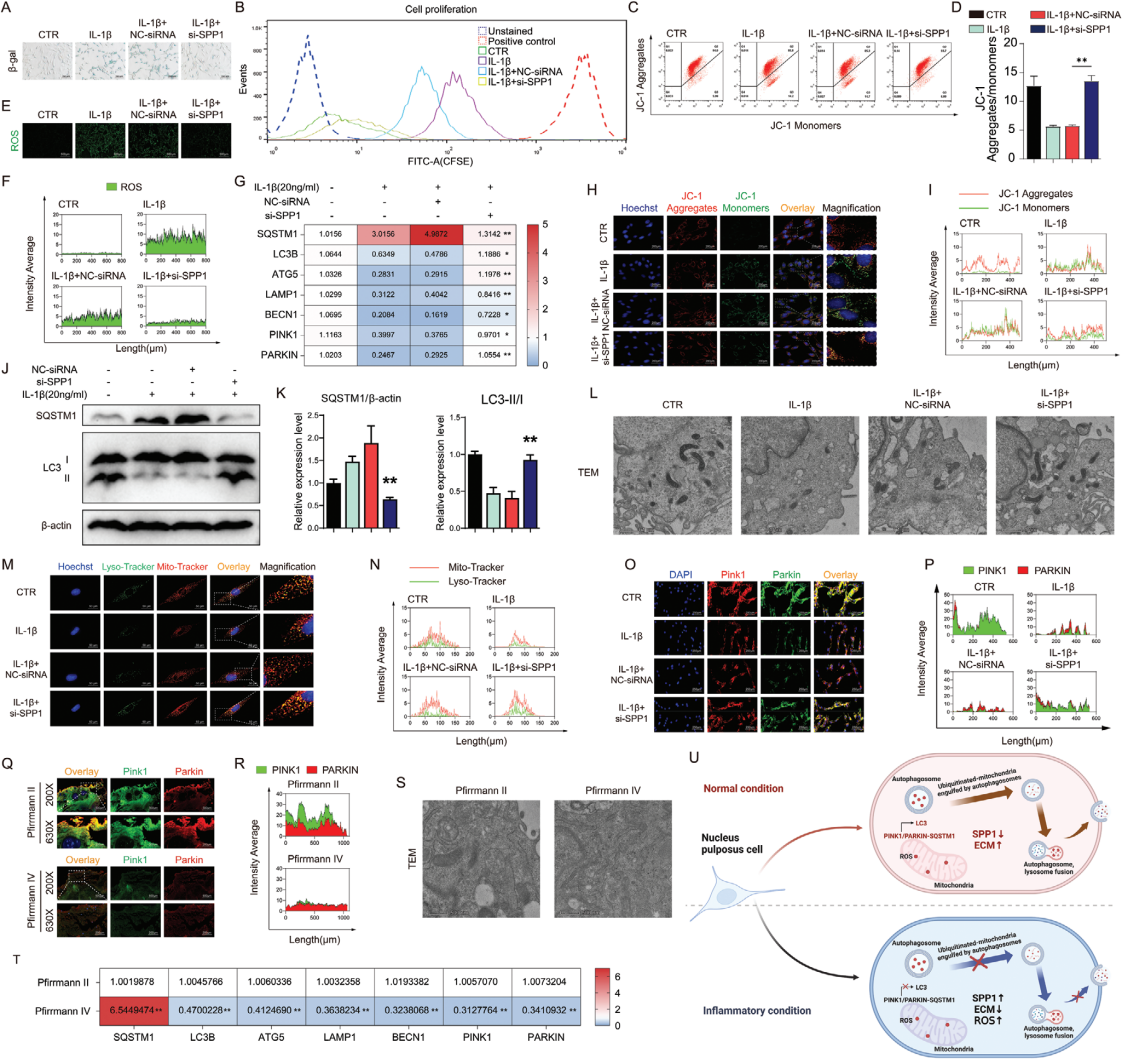
SPP1 induced senescence through ROS accumulation mediated by mitophagy reduction in NPs. A) β‐gal staining, 250 µm, *n* = 3; B) Flow cytometry (CFSE‐FITC) of cell proliferation, *n* = 3; C,D) JC‐1 flow cytometry, *n* = 3; E,F) Representative fluorescence images of ROS, 500 µm, *n* = 3; G) Relative mRNA level of SQSTM1, LC3B, ATG5, LAMP1, BECN1, PINK1 and PARKIN of NPs, *n* = 6; H,I) Detection of JC‐1 aggregates (red) and monomers (green) in NPs by confocal fluorescence microscopy, 200 µm, *n* = 3; J,K) Protein expression of SQSTM1 and LC3 II/I, *n* = 3; L) Representative TEM images of NPs, 1 µm, *n* = 3; M,N) Colocalization of lysosome (green) and mitochondria (red) by confocal fluorescence microscopy, 50 µm, *n* = 3; O,P) Representative immunofluorescence images of PINK1 and PARKIN, 250 µm, *n* = 3; Q,R) Representative immunofluorescence images of PINK1 and PARKIN, 500 µm, 200 µm, *n* = 5; S) Representative TEM images of nucleus pulposus tissue (Pfirrmann II and Pfirrmann IV), 500 nm, *n* = 5; T) Relative mRNA level of SQSTM1, LC3B, ATG5, LAMP1, BECN1, PINK1, and PARKIN of nucleus pulposus tissue (Pfirrmann II and Pfirrmann IV), *n* = 5; U) SPP1 promoted osteogenic differentiation of nucleus pulposus cells and disrupted extracellular matrix homeostasis under inflammatory conditions, the picture was drawn by Biorender (https://app.biorender.com/). Means ± S.E.M. ^*^ *p* < 0.05, ^**^ *p* < 0.01 versus IL‐1β+NC‐siRNA & versus Pfirrmann II.

To investigate the cause for disturbance of extracellular matrix and osteogenesis of inflammatory NPs induced by IL‐1β, cell senescence was detected to explore the potential mechanism first. After SPP1 knockdown, SA‐β‐gal staining performed a lighter staining, indicative of alleviation of senescence (**Figure** **4**A). The results of flow cytometry (CFSE‐FITC) suggested that knockdown of SPP1 significantly elevated NPs proliferation (Figure 4B). ROS in NPs also decreased due to SPP1 knockdown (Figure 4E,F). To examine whether mitochondria damage was one of the causes of ROS high production, JC‐1 flow cytometry revealed JC‐1 monomers decreased in SPP1 knockdown NPs, while JC‐1 aggregates increased (Figure 4C,D). In line with flow cytometry images, fluorescence analysis of JC‐1 also verified these results, in which green fluorescence signal was enhanced and diffused outside the mitochondria with insignificant reduction of red fluorescence signal in the mitochondria in SPP1 knockdown NPs (Figure 4H,I). These suggested that SPP1 knockdown might improve mitochondria functions, such as mitophagy, which contributed to ROS clearance. Expression of LC3B, ATG5, LAMP1, BECN1, PINK1, and PARKIN mRNA as well as LC3 II/I, PINK1, and PARKIN protein increased in SPP1 knockdown NPs, while SQSTM1 protein and mRNA expression decreased (Figure 4G,J,K,O,P). Further detection of colocalization of mitochondria and lysosomes indicated that SPP1 knockdown increased their interaction in NPs induced by IL‐1β (Figure 4M,N). Additionally, TEM was performed to evaluate mitophagy. Compared with mitochondrial swelling, vacuolation, and structural destruction induced by IL‐1β in NPs, knockdown of SPP1 improved mitochondria structure and mitophagy, and promoted the clearance of damaged mitochondria (Figure 4L). Similarly, nucleus pulposus tissue of Pfirrmann II and Pfirrmann IV were examined for TEM and results verified the above results (Figure 4S). Expression of LC3B, ATG5, LAMP1, BECN1, PINK1, and PARKIN mRNA as well as PINK1 and PARKIN protein decreased in Pfirrmann IV nucleus pulposus tissue, while SQSTM1 mRNA expression increased (Figure 4Q–T). This indicated an increased expression of SPP1, inhibited mitophagy, abnormally accumulated ROS, and disordered extracellular matrix in degenerative NPs (Figure 4U).

### 2.5 SPP1 Knockdown Attenuated Matrix Disturbance and Calcification Tendency by Mitophagy Activation in Intervertebral Discs

**Figure 5:**
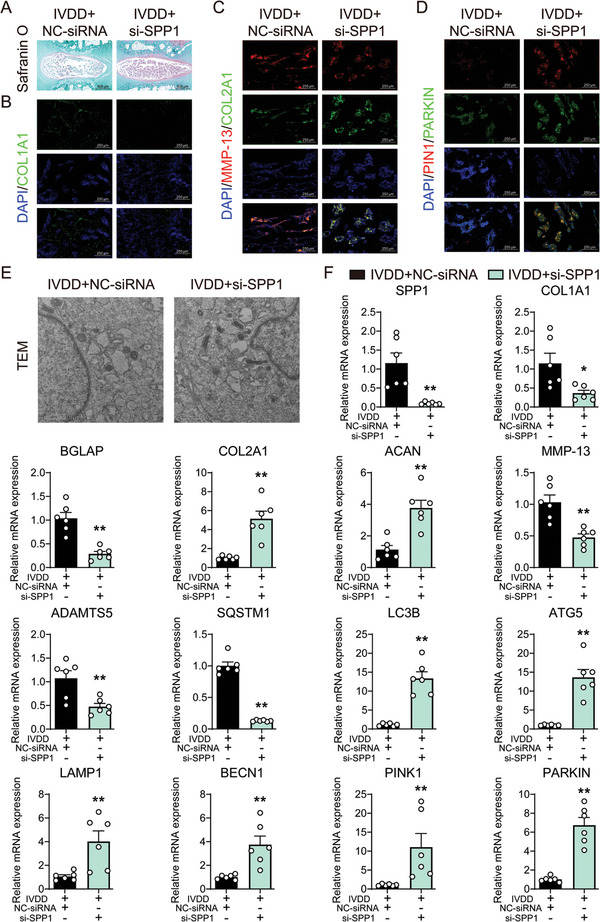
SPP1 knockdown attenuated matrix disturbance and calcification tendency by mitophagy activation in intervertebral discs. A) Safranin O staining, 500 µm, *n* = 5; B) Representative immunofluorescence images of COL1A1, 250 µm, *n* = 5; C) Representative immunofluorescence images of COL2A1 and MMP13, 250 µm, *n* = 5; D) Representative immunofluorescence images of PINK1 and PARKIN, 250 µm, *n* = 5; E) Representative TEM images of the rat nucleus pulposus tissue, 1 µm, *n* = 3; F) Relative mRNA level of SPP1, BGLAP, CLO1A1, ACAN, COL2A1, MMP13, ADAMTS5, SQSTM1, LC3B, ATG5, LAMP1, BECN1, PINK1, and PARKIN, *n* = 6. ^*^ *p* < 0.05, ^**^ *p* < 0.01 versus IVDD+NC‐siRNA.

We further evaluated the therapeutic potential of SPP1 knockdown on degeneration of nucleus pulposus. Upon siRNA‐mediated SPP1 knockdown, the structural damage and morphological abnormalities of nucleus pulposus were improved, and Safranin O staining got darker (**Figure** **5**A), which indicated the synthesis of glycosaminoglycan in extracellular matrix was promoted by SPP1 knockdown. The mRNA expression of SPP1, osteogenesis markers (BGLAP and COL1A1) and extracellular matrix degradation markers (ADAMTS5 and MMP13) decreased after in SPP1‐knockdown nucleus pulposus tissue, while markers of matrix synthesis (ACAN and COL2A1) increased (Figure 5F). Similarly, immunofluorescence of COL1A1 and MMP13 performed decreased intensity, and COL2A1 fluorescence intensity increased (Figure 5B,C). Further detection of TEM indicated improved mitochondria structure and mitophagy, and promoted damaged mitochondria clearance in SPP1 knockdown nucleus pulposus tissue, compared with IVDD group (Figure 5E). Mitophagy mediated by PINK1/PARKIN in ubiquitin‐dependent pathways was further examined. Immunofluorescence of PINK1 and PARKIN performed increased intensity in SPP1‐knockdown nucleus pulposus (Figure 5D). The mRNA expression of LC3B, ATG5, LAMP1, BECN1, PINK1, and PARKIN increased, while SQSTM1 decreased after in SPP1‐knockdown nucleus pulposus tissue (Figure 5F).

### 2.6 SPP1‐ITGα5/β1 Signaling Promoted Osteogenesis and Extracellular Matrix Homeostasis Disruption of NPs

**Figure 6:**
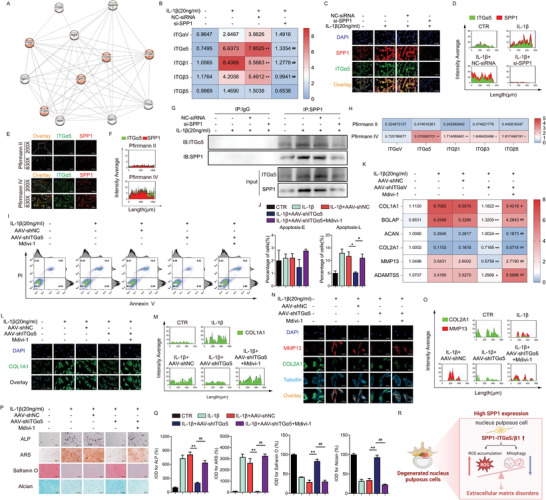
SPP1‐ITGα5/β1 signaling promoted osteogenesis and extracellular matrix homeostasis disruption of NPs. A) Protein interaction network analysis; B) Relative mRNA level of ITGαV, ITGα5, ITGβ1, ITGβ3 and ITGβ5 of NPs, *n* = 6; C,D) Representative immunofluorescence images of SPP1 and ITGα5 of NPs, 250 µm, *n* = 3; E,F) Representative immunofluorescence images of SPP1 and ITGα5 of nucleus pulposus tissue (Pfirrmann II and Pfirrmann IV), 500 µm, 200 µm, *n* = 3; G) Co‐immunoprecipitation assays, *n* = 3; H) Relative mRNA level of ITGαV, ITGα5, ITGβ1, ITGβ3, and ITGβ5 of nucleus pulposus tissue (Pfirrmann II and Pfirrmann IV), *n* = 5; I,J) Flow cytometry of apoptosis, *n* = 3; K) Relative mRNA level of COL1A1, BGLAP, ACAN, COL2A1, MMP13, and ADAMTS5, *n* = 6; L,M) Representative immunofluorescence images of COL1A1, 400×, *n* = 3; N,O) Representative immunofluorescence images of COL2A1 and MMP13, 630×, *n* = 3; P,Q) ALP staining, ARS staining, Safranin O staining and alcian staining, 200×, *n* = 3; R) ITGα5 induced senescence through ROS accumulation mediated by mitophagy reduction in NPs, the picture was drawn by Biorender (https://app.biorender.com/). Means ± S.E.M. ^*^ *p* < 0.05, ^**^ *p* < 0.01 versus IL‐1β+NC‐siRNA & versus Pfirrmann II & AAV‐shNC, ^#^ *p* < 0.05, ^##^ *p* < 0.01 versus IL‐1β+si‐SPP1 & versus IL‐1β+AAV‐shITGα5.

Single‐cell RNA‐seq analysis revealed IVDD mediated by upregulation of inflammatory response NP cells and fibrocartilaginous NP cells, in which SPP1 exerted crucial functions along with integrin receptor family.^[^ ^16^ ^]^ Protein interaction network analysis was performed on the STRING website (https://cn.string‐db.org/) for SPP1 and various subunits of the integrin receptor family (**Figure** **6**A). Upon SPP1 knockdown, RT‐qPCR and immunofluorescence were performed, indicative of significant decreases of ITGα5, ITGβ1 and ITGβ3 mRNA expression (Figure 6B), and indicative of SPP1 and ITGα5 protein expression decreases (Figure 6C,D). In Pfirrmann IV nucleus pulposus, ITGα5 and SPP1 fluorescence intensity increased significantly (Figure 6E,F) along with increased mRNA expression of ITGα5, ITGβ1, ITGβ3 and ITGβ5 (Figure 6H). Co‐immunoprecipitation assays revealed protein‐protein interaction between SPP1 and ITGα5 (Figure 6G). Since the integrins were α/β heterodimer, ITGα5 was selected for the next mechanistic study,^[^ ^14^ ^]^ and the efficiency of ITGα5 knockdown was verified (Figure S2, Supporting Information). Further, flow cytometry revealed that AAV‐shRNA‐mediated knockdown of ITGα5 in NPs significantly alleviated apoptosis caused by IL‐1β, especially in the late (end‐stage) apoptotic cells (Annexin V–FITC^+^/PI^+^) (Figure 6I,J). Upon ITGα5 knockdown, the mRNA expression of osteogenesis markers (BGLAP and COL1A1) and extracellular matrix degradation markers (ADAMTS5 and MMP13) decreased, while markers of matrix synthesis (ACAN and COL2A1) increased (Figure 6K). The ALP staining (3 days induction) and ARS staining (10 days induction) revealed that knockdown of ITGα5 exhibited substantial osteogenesis decreases of NPs under inflammatory conditions (Figure 6P,Q). Further, safranin O staining and alcian staining were conducted to confirm that degeneration of NPs in the inflammatory state has been alleviated, manifested as ITGα5 knockdown enhanced staining, indicating increased glycosaminoglycan and mucopolysaccharide synthesis (Figure 6P,Q). Immunofluorescence of COL1A1 and MMP13 performed decreased intensity, and COL2A1 fluorescence intensity increased, which further proved the above findings (Figure 6L–O).

### 2.7 ITGα5 Induced Senescence Through ROS Accumulation Mediated by Mitophagy Reduction in NPs

**Figure 7:**
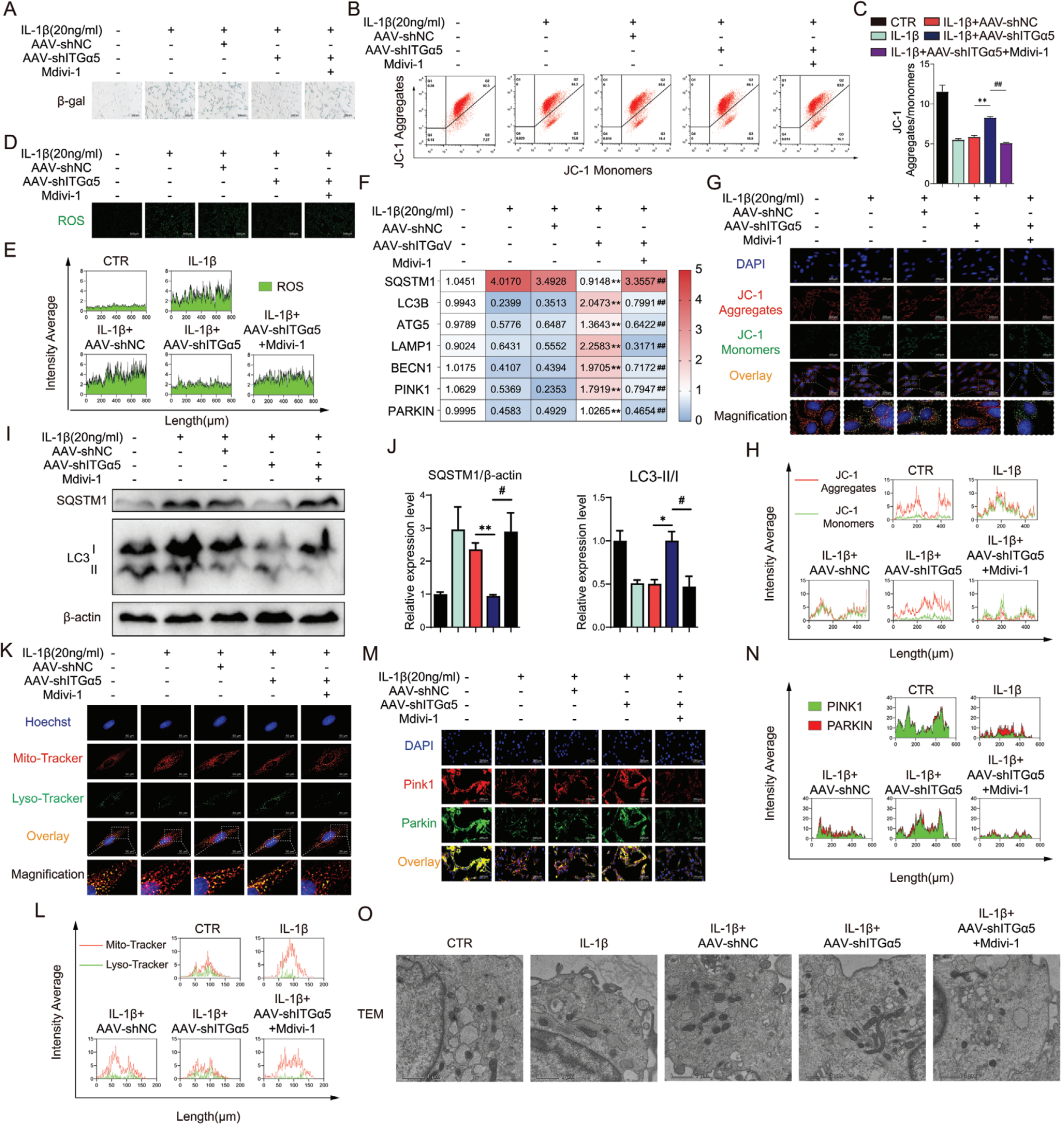
ITGα5 induced senescence through ROS accumulation mediated by mitophagy reduction in NPs. A) β‐gal staining, 250 µm, *n* = 3; B,C) JC‐1 flow cytometry, *n* = 3; D,E) Representative fluorescence images of ROS, 500 µm, *n* = 3; F) Relative mRNA level of SQSTM1, LC3B, ATG5, LAMP1, BECN1, PINK1 and PARKIN, *n* = 6; G,H) Detection of JC‐1 aggregates (red) and monomers (green) in NPs by confocal fluorescence microscopy, 200 µm, *n* = 3; I,J) Protein expression of SQSTM1 and LC3 II/I, *n* = 3; K,L) Colocalization of lysosome (green) and mitochondria (red) by confocal fluorescence microscopy, 50 µm, *n* = 3; M,N) Representative immunofluorescence images of PINK1 and PARKIN, 250 µm, *n* = 3; O) Representative TEM images of NPs, 1 µm, *n* = 3. Means ± S.E.M. ^*^ *p* < 0.05, ^**^ *p* < 0.01 versus IL‐1β+NC‐siRNA & versus Pfirrmann II.

After ITGα5 knockdown, SA‐β‐gal staining performed a lighter staining, indicative of alleviation of senescence (**Figure** **7**A). ROS in NPs decreased due to ITGα5 knockdown (Figure 7D,E). To examine the role of ITGα5 on mitochondria function, JC‐1 flow cytometry was performed to reveal JC‐1 monomers decreased in ITGα5 knockdown NPs, while JC‐1 aggregates increased (Figure 7B,C). In line with flow cytometry images, fluorescence analysis of JC‐1 also verified these results, in which green fluorescence signal was enhanced and diffused outside the mitochondria with insignificant reduction of red fluorescence signal in the mitochondria in ITGα5 knockdown NPs (Figure 7G,H). These suggested that ITGα5 knockdown improved mitochondria functions, such as mitophagy, which contributed to ROS clearance (Figure 6R). Expression of LC3B, ATG5, LAMP1, BECN1, PINK1, and PARKIN mRNA as well as LC3B II/I, PINK1, and PARKIN protein increased in ITGα5 knockdown NPs, while SQSTM1 mRNA and protein expression decreased (Figure 7F,I,J,M,N). Further detection of colocalization of mitochondria and lysosomes indicated that ITGα5 knockdown increased their interaction in NPs induced by IL‐1β (Figure 7K,L). Additionally, TEM was performed to evaluate mitophagy. Compared with mitochondrial swelling, vacuolation, and structural destruction induced by IL‐1β in NPs, knockdown of ITGα5 improved mitochondria structure and mitophagy, and promoted the clearance of damaged mitochondria (Figure 7O).

### 2.8 SPP1‐ITGα5/β1 Signaling Promoted Osteogenesis and Extracellular Matrix Homeostasis Disruption in Degenerative NPs by Impairing Mitophagy

To further demonstrate that SPP1‐ITGα5/β1 signaling exerted functions by impairing mitophagy in degenerative NPs, NPs in an inflammatory state were treated by Mdivi‐1, which was well‐established as a potent mitophagy inhibitor.^[^ ^19^ ^]^ The alleviation of apoptosis induced by ITGα5 knockdown was blocked by Mvidi‐1 (Figure 6I,J). The mRNA expression of osteogenesis markers (COL1A1 and BGLAP) decreased upon Mdivi‐1 treatment (Figure 6K), and ALP staining (3 days induction) and ARS staining (10 days induction) performed darker staining and numerous mineralized nodules (Figure 6P,Q). Further detection of extracellular matrix synthesis and degradation revealed a decrease of COL2A1 and ACAN mRNA expression and an increase mRNA expression of MMP13 and ADAMTS5 (Figure 6K), along with lighter Safranin O staining and alcian staining, which indicated glycosaminoglycan and mucopolysaccharide synthesis was hindered upon Mdivi‐1 treatment (Figure 6P,Q). Additionally, affected by Mdivi‐1, COL1A1, and MMP13 protein expression increased, and COL2A1 deceased (Figure 6L–O).

Furthermore, the attenuation of NPs senescence induced by ITGα5 knockdown was partially reversed by Mdivi‐1 (Figure 7A). Upon Mdivi‐1 treatment with ITGα5 knockdown in degenerative NPs, JC‐1 monomers increased and JC‐1 aggregates decreased (Figure 7B,C), with enhanced green fluorescence signal of JC‐1 monomers diffusing outside the mitochondria, along with reduced red JC‐1 aggregates fluorescence signal (Figure 7G,H). This also explains why the fluorescence signal of ROS was enhanced after Mdivi‐1 treatment (Figure 7D,E). Expression of LC3B, ATG5, LAMP1, BECN1, PINK1, and PARKIN mRNA as well as LC3 II/I, PINK1, and PARKIN protein decreased, while SQSTM1 mRNA and protein expression increased (Figure 7F,I,J,M,N). Further detection of colocalization of mitochondria and lysosomes indicated that Mdivi‐1 decreased their interaction in ITGα5‐knockdown NPs (Figure 7K,L). Additionally, compared with relatively normal, regular, and complete morphology of mitochondria in after ITGα5 knockdown, Mdivi‐1 hindered these effects, leading to mitochondrial swelling, vacuolation, and structural destruction again (Figure 7O), indicating that Mdivi‐1 obstructed the improvement of mitophagy and the maintenance of normal mitochondrial morphology induced by ITGα5 knockdown.

### 2.9 ITGα5 Knockdown Attenuated Matrix Disturbance and Calcification Tendency by Mitophagy Activation in Intervertebral Discs

**Figure 8:**
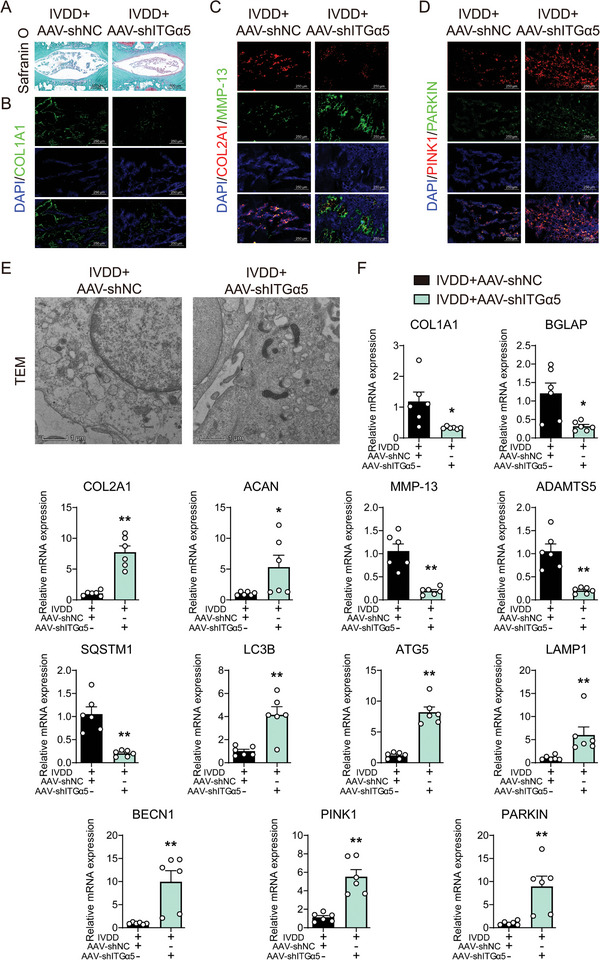
ITGα5 knockdown attenuated matrix disturbance and calcification tendency by mitophagy activation in intervertebral discs. A) Safranin O staining, 100×, *n* = 6; C) Relative mRNA level of BGLAP, CLO1A1, ACAN, COL2A1, MMP13, and ADAMTS5, *n* = 6; B) Representative immunofluorescence images of COL1A1, COL2A1 and MMP13, PINK1, and PARKIN, 400×, *n* = 3; (D) Representative TEM images of the rat nucleus pulposus tissue, 14 000×, *n* = 3; E) Relative mRNA level of SQSTM1, LC3B, ATG5, LAMP1, BECN1, PINK1 and PARKIN, *n* = 6. ^*^ *p* < 0.05, ^**^ *p* < 0.01 versus IVDD+AAV‐shNC.

We further evaluated the therapeutic potential of ITGα5 knockdown on degeneration of nucleus pulposus. Upon siRNA‐mediated ITGα5 knockdown, the structural damage and morphological abnormalities of the nucleus pulposus were ameliorated, accompanied by a darker Safranin O staining (**Figure** **8**A), indicating enhanced glycosaminoglycan synthesis in the extracellular matrix due to ITGα5 knockdown. The mRNA expression of osteogenesis markers (BGLAP and COL1A1) and extracellular matrix degradation markers (ADAMTS5 and MMP13) decreased following ITGα5‐knockdown in nucleus pulposus tissue, while markers of matrix synthesis (ACAN and COL2A1) increased (Figure 8C). Similarly, immunofluorescence of COL1A1 and MMP13 exhibited decreased intensity, while COL2A1 fluorescence intensity increased (Figure 8B,C). Further examination using TEM indicated improved mitochondria structure and mitophagy, as well as promoted clearance of damaged mitochondria, reduced mitochondrial ridge swelling, and enhanced mitochondrial biogenesis in the ITGα5 knockdown nucleus pulposus tissue compared to the IVDD group (Figure 8E). Mitophagy mediated by PINK1/PARKIN in ubiquitin‐dependent pathways was also investigated. Immunofluorescence of PINK1 and PARKIN showed increased intensity in the ITGα5‐knockdown nucleus pulposus (Figure 8D). Additionally, the mRNA expression of LC3B, ATG5, LAMP1, BECN1, PINK1, and PARKIN increased, while SQSTM1 decreased after in SPP1‐knockdown nucleus pulposus tissue (Figure 8F).

## 3 Discussion

**Figure 9:**
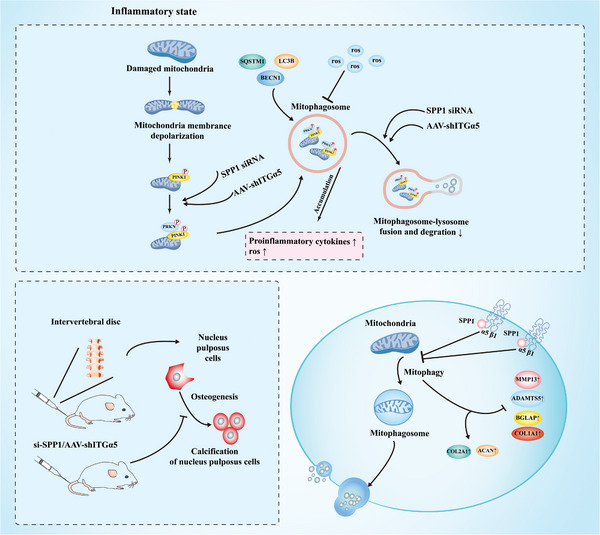
Mitophagy inhibitor SPP1 regulates nucleus pulposus cell fate during degeneration and calcification by activating ITGα5/β1 and inhibiting ubiquitin‐dependent PINK1/PARKIN pathway. The figure was drawn by 58pic (https://www.58pic.com).

This study demonstrated that SPP1‐ITGα5/β1 drives nucleus pulposus degeneration and calcification in human and rat degenerative intervertebral discs. We first found that SPP1 as well as COL1A1 increased in Pfirrmann IV human IVD, suggesting that highly degenerative nucleus pulposus tissues had considerable calcification potential, which was associated with SPP1, and similar results were found in the rat needle model. Further, it was revealed that the activation of SPP1 enhanced the ITGα5/β1 function, which inhibited the synthesis and promoted the degradation of extracellular matrix and was involved in calcium deposition and mineralization in NPs. From this, we conclude that inhibiting the deleterious effects of SPP1‐ITGα5/β1 is a potential therapeutic intervention for IVDD (**Figure** **9**).

The most typical symptom of IVDD is pain caused by nerve compression.^[^ ^20^ ^]^ The way to relieving pain in a short time is surgical treatment, including removal of nucleus pulposus, decompression and fusion fixation, etc.^[^ ^21^ ^]^ However, surgical treatment often has a certain degree of trauma, accompanied by possible complications and other potential risks, including intraoperative or postoperative infection, spinal cord injury, wound nonunion, etc.^[^ ^21^, ^22^ ^]^ Therefore, it is particularly important to find new and reliable methods for the prevention and treatment of IVDD. In IVDD process, calcification is often accompanied, which contributes to aggravated compression of nerves and pain of patients.^[^ ^20^ ^]^ Nevertheless, it has not been demonstrated that there is a pre‐reconnection between nucleus pulposus degeneration and calcification during IVDD. In this study, we demonstrated that the calcification potential of nucleus pulposus was increased along with the degeneration IVDD, and part of nucleus pulposus extracellular matrix was mineralized and calcium salt was deposited, which correlated with the increased expression of SPP1 in degenerative nucleus pulposus tissues.

Certainly, similar to OA, IVDD is a complex degenerative disease and induced by a variety of inducing factors, such as chronic inflammation, aging, oxidative stress, and ECM disorder of nucleus pulposus cells caused by long‐term mechanical load and severe trauma.^[^ ^23^ ^]^ Our data demonstrated that SPP1 led to senescence of NPs. Cellular senescence is often closely associated with mitochondrial dysfunction and chronic inflammation.^[^ ^24^ ^]^ The abnormal accumulation of ROS is involved in the occurrence of chronic inflammation.^[^ ^25^ ^]^ In our study, knockdown of SPP1 partially ameliorated the apoptosis of NPs under inflammatory conditions, and also reduced the intracellular ROS level. ROS scavenging is related to mitochondrial function, and mitochondrial dysfunction and mitophagy inhibition often led to ROS scavenging disorders and abnormal accumulation.^[^ ^26^ ^]^ In our study, SPP1 knockdown ameliorated the reduction of mitochondrial membrane potential and inhibition of mitophagy in inflammatory NPs, which contributed to ROS clearance.

In previous studies, integrin family is often considered as the effector molecules of intercellular signal transduction and cell adhesion.^[^ ^27^ ^]^ Furthermore, integrins are not only involved in cell‐cell signaling, but also in glucose uptake and the connection of extracellular matrix to cytoskeleton.^[^ ^28^ ^]^ Recent studies have found that integrins can be internalized into the endocytosis pathway and then recycled back to the plasma membrane to regulate other functions in cells.^[^ ^29^ ^]^ Moreover, the function of integrins is also involved in the control of intracellular signal transduction, and participates in pathological processes such as neurodegeneration, tissue fibrosis and cancer.^[^ ^30^ ^]^ We identified ITGα5/β1 in a single‐cell sequencing analysis, acting downstream of SPP1 and upstream of PINK1/PARKIN in degenerative NPs.^[^ ^16^ ^]^ ITGα5/β1 is an integrin involved in intercellular and intracellular signal transduction.^[^ ^31^ ^]^ Since the previous studies on its biological function and pathophysiological function of various diseases are relatively few, its specific function and mechanism remain to be further explored. It has been found that ITGα5/β1 promoted the production of nitric oxide to inhibit the apoptosis of rBMSCs and promote the differentiation of HPASMC.^[^ ^32^ ^]^ Moreover, ITGα5/β1 has been regarded to promote mesothelial cell proliferation and peritoneal metastasis of ovarian cancer cells.^[^ ^33^ ^]^ Our results suggested that SPP1‐ITGα5/β1 is an intracellular signal transduction pathway, in which SPP1 in degenerative NPs mediated intracellular mitochondrial dysfunction and inhibits mitophagy by activating ITGα5/β1, accelerating cellular aging, which in turn affects extracellular matrix homeostasis.

PINK1/PARKIN is a classic ubiquitin‐dependent mitophagy pathway, in which LC3‐positive autophagosomes are recruited by the ubiquitin‐proteasome system and bridge to ubiquitinated mitochondria, which are subsequently engulfed and fused with lysosomes for substrate degradation.^[^ ^34^ ^]^ In this process, SQSTM1 accumulates in mitochondria and is recruited by PARKIN to mediate the binding of ubiquitylated substrates to LC3.^[^ ^35^ ^]^ Interestingly, our results demonstrated that SQSTM1 expression decreased after SPP1‐ITGα5/β1 knockdown, while PINK1/ Parkin‐mediated mitophagy was promoted. We hypothesized that the recruitment of SQSTM1 on damaged mitochondria does not represent its increased intracellular expression, but rather that the decrease of SQSTM1 accompanied by increased LC3B expression reflects the unobstructed autophagic flow in the cells. This further indicated that SPP1 inhibited mitophagy in NPs, leading to mitochondrial dysfunction, thereby causing cellular senescence.

Previous studies have demonstrated that the activation of PINK1/PARKIN ubiquitin‐dependent mitophagy is affected by multiple epigenetic alterations, such as, PINK1 autophosphorylation at Ser228, PINK1 ubiquitin phosphorylation at Ser65, and phosphorylation of Parkin by PINK1 at Ser65.^[^ ^36^ ^]^ Additionally, ubiquitination ligases (cytoplasmic E3 ubiquitin ligases), PARKIN substrates (mitofusin and voltage‐dependent anion‐selective channel protein 1), and TBK1 affect the normal function of mitophagy.^[^ ^37^ ^]^ As we have demonstrated that SPP1‐ITGα5/β1 inhibits PINK1/PARKIN activation in NPs to suppress mitophagy, more detailed mechanisms require further investigation.

Nevertheless, there are some limitations in this study: we used a preventive protocol rather than a therapeutic protocol. Due to the limitations of the intervertebral disc degeneration model,^[^ ^38^ ^]^ repeated intervertebral disc acupuncture will damage the nucleus pulposus tissue repeatedly, which may obscure the therapeutic effect of gene intervention. Therefore, we do not recommend secondary viral injection in IVDD animals to explore therapeutic effects of SPP1 or ITGa5 knockdown, which is contrary to our intention to explore therapeutic strategies for disc degeneration. In addition, we genetically engineered the nucleus pulposus tissue directly in situ, causing harm to the disc itself, even though our positive control group also underwent disc acupuncture. In future studies, we hope to explore milder and more stable prevention and therapeutic strategies, such as non‐invasive nano‐drug delivery systems, sustained drug delivery systems, and small molecule drugs with bioactive ingredients similar to the existing treatment strategies with certain feasibility.^[^ ^39^ ^]^

In summary, we have revealed that SPP1‐ITGα5/β1 plays crucial role in degeneration and calcification in human and rat degenerative IVD that are induced by NPs senescence mediated by mitophagy inhibition. Our results provide important insights into the role of SPP1 in nucleus pulposus calcification in IVDD by inducing nucleus pulposus cell senescence through inhibition of mitophagy, and may help develop potential new strategies for IVDD treatment.

## 4 Experimental Section

### Chemicals and Reagents

Isoflurane was purchased from Baxter Healthcare Co. (Deerfield, IL, America). The antibodies of COL1A1 (A16891), COL2A1 (A19308), MMP13 (A11755), SPP1 (A1361), goat anti‐rabbit IgG (AC005), FITC goat anti‐rabbit IgG (H+L) (AS011) and cy3 goat anti‐rabbit IgG (H+L) (AS007) were purchased from ABclonal Technology Co., Ltd. (Wuhan, China). The antibodies of SPP1 (22952‐1‐AP), PTEN‐induced kinase 1 (PINK1, 23274‐1‐AP) and PARKIN (14060‐1‐AP) were purchased from Proteintech Group Inc. (Wuhan, China), and ITGα5 (14‐0493‐81 and PA5‐79529) was purchased from Thermo Fisher Scientific Inc. The total RNA TRIzol kit was purchased from Omega Bio‐Tek (Doraville, Georgia, America). FITC‐Phalloidin (CA1620) and TRITC‐Phalloidin (CA1610) were purchased from Solarbio Science & Technology (Beijing) Co., Ltd. Reverse transcription and real‐time quantitative polymerase chain reaction (RT‐qPCR) kits (Q223) were purchased from Takara Biotechnology Co., Ltd. (Dalian, China). All the primers were synthesized by TIANYIHUIYUAN Biotechnology Co., Ltd. (Wuhan, China). The other reagents for experiments were of analytical grade.

### Isolation of Human Nucleus Pulposus Tissues

An endoscopic surgical approach during spinal orthopedics was used to surgically extract human nucleus pulposus tissues from intervertebral discs (ethics code: No. KYLL‐2022(ZM)‐1051). These samples were subsequently processed in a sterilized environment, including removal of extraneous AF and ligamentum flavum. The isolated human nucleus pulposus tissues were then immersed in PBS for 5 min before being transferred to a Petri dish containing complete medium (DMEM/F12 medium containing 10% FBS and 1% penicillin‐streptomycin) and cultured for further experiments.

### Animals and Treatment

SPF Wistar rats [certification number: No. 20231113Aazz0619000875, license number: SYXK (Lu) 2023‐0003] weighing 200 ± 20 g (males) were obtained from the Beijing Vital River Laboratory Animal Technology Co., Ltd. (Beijing, China). Animal experiments were handled in the model animal research center of Shandong University (Jinan, China). All animal experiments complied with the National Research Council's Guide for the Care and Use of Laboratory Animals. Furthermore, the 3Rs principles were implemented to eliminate unnecessary harm to animals used in research.

Wistar rats were housed in cages with wire‐mesh floors in an air‐conditioned room under standard conditions and allowed a normal diet. All rats were acclimated 1 week before experimentation. Animal surgical modeling was according to the previous studies.^[^ ^38^, ^40^ ^]^ Thirty‐six rats were randomly divided into sham surgical group (*n* = 6), IVDD group (*n* = 6), IVDD+NC‐siRNA group (*n* = 6), IVDD+si‐SPP1 group (*n* = 6), IVDD+AAV‐shNC group (*n* = 6), and IVDD+AAV‐shITGα5 group (*n* = 6). In the latter four groups of rats, in vivo siRNA (NC‐siRNA and si‐SPP1, Ribobio, China) of 100 nmol kg^−1^ and AAV2‐shRNA (AAV‐shNC and AAV‐shITGα5, Haixing Biosciences, China) of 1 × 10^10^ viral genomes were injected at the same time as acupuncture was applied to the rat caudal disc to ensure that the IVDD received treatment. The rats were sacrificed and their caudal vertebrae were taken for subsequent histological examination to assess the degree of IVDD after the intended treatment regimen.

### Histochemical Hematoxylin and Eosin (H&E) Staining, Safranin O Staining, and ARS Staining

After fixation in 4% paraformaldehyde, rat tail vertebrae were decalcified using 14% EDTA (pH 7.4; Servicebio; China) at 37 °C for 28 days, followed by gradient dehydration and paraffin‐embedded. In contrast, human nucleus pulposus tissues can be fixed for subsequent processing without decalcification. The fixed rat tail vertebrae were sectioned in coronal view. Sections (4‐µm thick) were rehydrated and applied with HE staining, and the neighboring slice was subjected to safranin‐O staining and ARS staining. The sections were observed and photographed with an Olympus IX73 inverted microscope (Olympus, Tokyo, Japan). Finally, relevant examination indexes were evaluated from each photomicrograph (*n* = 6). All evaluations were performed blinded with an image analysis system (Olympus, Tokyo, Japan).

### Immunofluorescence Staining of Rat and Human Nucleus Pulposus Tissues

Paraffin‐embedded tissues were cut into 4 µm sections, deparaffinized in xylene, and rehydrated. Samples were boiled in citrate buffer (10 mm, pH 6.0) for 3 min and rinsed three times with PBS. After blocking in 5% BSA for 20 min at room temperature, the sections were then incubated overnight at 4 °C with the primary antibody, followed by Tyramide signal amplification (TSA; Recordbio; China) solution following the manufacturer's protocol for 10 min, followed by subsequent incubation with the fluorescent secondary antibody (1:200) for 2 h at room temperature. The procedure was then repeated for incubation with the second primary and secondary antibody, and stain the sections with 4′,6‐diamidino‐2‐phenylindole (DAPI) (1 µg mL^−1^) for 15 min. In this study, double immunofluorescence labeling was performed for COL1A1 (1:100) and SPP1 (1:100), for COL2A1 (1:100) and MMP13 (1:100), and for PINK1 (1:100) and PARKIN (1:100). Additional sections were incubated with nonspecific IgG instead of the primary antibody to act as negative controls. Fluorescence images were captured using an Olympus IX73 inverted microscope (Olympus, Tokyo, Japan). The fluorescence density of the genes above were measured by Image J 1.52v analysis software according to the manufacturer's instructions.

### Isolation and Culture of Rat Nucleus Pulposus Cells

After anesthesia with isoflurane inhalation, the rats were euthanized, and subsequently, the intervertebral disc tissues of the rats were removed following the procedures described above. Subsequently, portions of the adjacent upper and lower vertebral bodies, the intact cartilage endplate, and the disc were separated. The isolated rat intervertebral disc tissues were washed in PBS for 5 min. After 30‐min 0.25% trypsin (Gibco, America) digestion and 6‐h 0.2% collagenase II (Solarbio, China) digestion, the cell suspension was re‐centrifuged, and the supernatant was removed. Then, the cells were blown up in DMEM/F12 medium (Gibco, America) containing 10% fetal bovine serum (Servicebio, China) and 1% penicillin‐streptomycin (Solarbio, China) and transferred to a new culture dish. As the degree of cell fusion reached 95%, the adherent cells were digested with 3 mL 0.25% trypsin and passaged at a ratio of 1:3. Next, NPs were seeded in 6‐well plates and 24‐well plates. When the degree of cell fusion reached 60–80%, the cells were treated with IL‐1β (20 ng mL^−1^, Abclonal, China) for 24 h to induce an inflammatory condition. All transfection experiments were performed using Lipofectamine 3000 (Thermo Fisher Scientific, L3000015) according to the manufacturer's instructions, and subsequent experiments were performed 30 h later. Transfection of AAV2‐shNC and AAV2‐shITGa5 was performed at 50% degree of cell fusion to ensure the effectiveness.

### Total RNA Extraction, Reverse Transcription, and RT‐qPCR

The nucleus pulposus tissues from rat and human were collected and total RNA was extracted from using TRIzol Reagent following the manufacturer's protocol, ground using zirconia magnetic beads. The cell samples were not ground. The total RNA was reverse transcribed using a first‐strand cDNA synthesis kit, and RT‐qPCR was performed using a SYBR Green qPCR Master Mix Kit and ABI StepOnePlus cycler (Applied Biosystems, Foster City, CA, AMERICA). The cycle threshold (Ct) was detected, and the relative expression of genes (SPP1, COL1A1, BGLAP, ACAN, COL2A1, MMP13, ADAMTS5, SQSTM1, LC3B, ATG5, LAMP1, BECN1, PINK1, PARKIN, ITGα5, ITGαV, ITGβ1, ITGβ3, ITGβ5) were determined using the 2‐△△Ct method with normalization to glyceraldehyde 3‐phosphatedehydrogenasefor (GAPDH) expression and used as a quantitative control.

### Western Blotting

NPs which were treated with different treatments were performed with protein extraction after the corresponding treatment time. The concentration of the extracted protein was detected by BCA methods. Western blotting protocols were performed as described in the previous studies.^[^ ^41^ ^]^ The grey values of target proteins (such as LC3 I, LC3 II, SQSTM1, and β‐actin) were determined by Image J software.

### Apoptosis Flow Cytometry

The NPs were inoculated into 12‐well plates and cultured for apoptosis analysis. Cells were collected and analyzed using the annexin V‐FITC/PI apoptosis detection kit (BD, America). Cells were washed twice by centrifugation (1000 rpm, 5 min, 4 °C) with PBS and re‐suspended in 400 µL 1X Annexin V binding solution. 5 µL of Annexin V‐FITC staining solution and 5 µL of PI staining solution were added to the cell suspension and incubated at 22 ± 1 °C in the dark for 20 min. Subsequently, Samples were immediately analyzed by FACS Calibur+Sort (BD, America). The data were analyzed with Flowjo X V10.0.7r2 software.

### CFSE Proliferation Assay

CFSE proliferation assays were performed following the instruction of CellTrace CFSE Cell Proliferation Kit (Thermo Fisher Scientific, C34554). Briefly, NPs were collected and stained in cell trace CFSE solution for 20 min at 37 °C, then distribute into culture plates. Three days after treatment accordingly, cells were harvested and analyzed using BD FACS Celesta (BD, America). Data were analyzed by Flowjo X V10.0.7r2 software.

### Osteogenic Differentiation and Treatment of Nucleus Pulposus Cells (ALP+ARS)

After administrated with diverse treatments, NPs were cultured in osteogenic differentiation medium (ODM; α‐MEM supplemented with 10% FBS, 100 nm dexamethasone, 10 mm beta‐glycerol phosphate, and 50 µm ascorbate‐2‐phosphate) to determine the osteogenic promoting function of SPP1 and ITGα5 in the IVD calcification. NPs were seeded on 24‐well plates at a density of 1 × 10^4^ per well and differentiated by ODM for 3 or 10 days. After 14 days of osteogenic differentiation, the cells were fixed in 4% FPA for 10 min, washed with PBS, and assayed with ALP Staining kit (Beyotime, China) following the manufacturer's instructions. After 14 days of osteogenic differentiation, NPs were fixed in 4% FPA for 10 min after washed by PBS, and then stained by 1% Alizarin Red Staining (ARS) solution (Beyotime) at pH 4.2 and 37 °C for 30 min. The total area of blue nodules in ALP and red nodules in ARS were detected by an Olympus IX73 inverted microscope (Olympus, Tokyo, Japan).

### Safranin O Staining and Alcian Staining

Safranin O staining and alcian staining were measured following the previous description.^[^ ^42^ ^]^ Briefly, NPs to be examined were washed with PBS and subsequently fixed with 4% paraformaldehyde for 20 min at room temperature and washed three times with PBS for 5 min each. The cells were then incubated with safranin O or alcian solution for 30 min at room temperature. After washing three times with PBS, safranin O staining and alcian staining were detected by an Olympus IX73 inverted microscope (Olympus, Tokyo, Japan). The integrated optical density of each sample was calculated using Image J 1.52v software.

### SA‐β‐Gal Staining

SA‐β‐gal activity was measured with a staining kit (Beyotime, China) following the manufacturer's protocols. The SA‐β‐gal levels of the samples were observed using Olympus IX73 inverted microscope (Olympus, Tokyo, Japan).

### ROS Determination

Intracellular ROS levels were measured using the ROS‐ID Total ROS detection kit (ENZO, America). Cells were seeded in six‐well plates and administrated with diverse treatments, ROS detection solution was added to the medium at a 1:1000 dilution with serum‐free medium and incubated at 37 ° C for 30 min. The ROS levels of the samples were observed using Olympus IX73 inverted microscope (Olympus, Tokyo, Japan). The average intensity of each sample was calculated using Image J 1.52v software.

### Mitochondrial Membrane Potential Determination

NPs were seeded on BeyoGold 35 mm confocal dishes (Beyotime, China) and six‐well plates for subsequent detection. Tetrechloro‐tetraethylbenzimidazol carbocyanine iodide (JC‐1, 10 µg mL^−1^; Beyotime, China) staining was performed at 37 °C for 20 min to determine NPs mitochondrial membrane potential as reported previously.^[^ ^43^ ^]^ The cell samples were detected using the Olympus SpinSR10 spinning disk confocal super‐resolution microscope (Olympus, Japan) and BD FACS Celesta (BD, America). Data were analyzed by Image J 1.52v software and Flowjo X V10.0.7r2 software.

### Transmission Electron Microscopy

Nucleus pulposus tissues and cells were fixed with 4% glutaraldehyde fixation buffer. The samples were subsequently postfixed, dehydrated, and embedded. Ultrathin sections were obtained using a Reichert ultramicrotome (Reichert‐Jung, Heidelberg, Germany) and stained with 1% uranyl lactate or lead citrate. Stained sections were viewed with a Field Emission Transmission Electron Microscope (Thermo Fisher Scientific, America).

### Detection of Mitochondrial and Lysosomal Colocalization

Removing the medium, NPs in the confocal dish were gently washed with PBS. Cells were cultured with DMEM/F12 medium containing MitoTracker (1:1000, M22426, Thermo Fisher Scientific, America) and LysoTracker (1:1000, L7528, Thermo Fisher Scientific, America) for 20 min at 37 ° C, washed three times with PBS, nuclei were stained with Hoechst (1:1000, 62 249, Thermo Fisher Scientific, America) for 20 min, and washed three times with PBS. The cells were observed by confocal microscopy. The cell samples were detected using the Olympus SpinSR10 spinning disk confocal super‐resolution microscope (Olympus, Japan). Data were analyzed by Image J 1.52v software.

### Immunofluorescence Staining of Nucleus Pulposus Cells

Nucleus pulposus cells were fixed at room temperature with 4% paraformaldehyde for 15 min after washing by pre‐cooled PBS, and the membrane was permeabilized by 0.5% Triton X‐100 for 15 min and the cells were blocked with 3% BSA at 22 ± 1 °C for 30 min without cleaning. NPs were incubated at 4 °C for 8–12 h with primary antibody, including COL1A1 (1:100 dilution respectively), followed by subsequent incubation with fluorescently labeled secondary antibody (1:100) of corresponding species at 22 ± 1 °C for 60 min after three times washing. Double immunofluorescence labeling was performed under the same TSA procedure for COL2A1 (1:100) and MMP13 (1:100), for PINK1 (1:100) and PARKIN (1:100) and for SPP1 (1:100) and ITGα5 (1:100), followed by subsequent incubation with phalloidin solution (5 µg mL) at 22 ± 1 °C for 1 h after three times washing. NPs were stained with 4′,6‐diamidino‐2‐phenylindole (DAPI; Thermo Fisher Scientific, AMERICA) for 5 min and then the cells were washed with PBS for five times (3–5 min per time). Finally, the cell samples were detected using the Olympus SpinSR10 spinning disk confocal super‐resolution microscope (Olympus, Japan).

### Protein‐Protein Interaction Network Analysis

The protein interaction network was analyzed by STRING website (https://cn.string‐db.org/). Input “# ITGα5 #ITGβ1 #ITGβ3 #ITGβ5 # ITGαV #CD44 #SPP1” in the protein name and select the Organisms as “Rattus norvegicus.” Then, all the searched proteins were selected by default for protein‐protein interaction network analysis.

### Co‐Immunoprecipitation (Co‐IP) Assay

Co‐immunoprecipitation assay was implemented in line with the previous description (31237474, 37095518). After washing with precooled PBS, the cells were transferred to 1.2 mL lysis buffer and lysed at −80 °C for 30 min. The samples were subsequently centrifuged at 13 000 rpm for 10 min at 4 °C and the supernatant was transferred to a new column. Samples were then divided into triplicate: the first for protein input and the other two using 1 µg of mock antibody IgG (1:100 dilution) as control or SPP1 antibody (1:1000 dilution) followed by incubation overnight at 4 °C with gentle shaking. Then, the samples were incubated with protein G magnetic beads for 6 h at 4 °C. After immunoprecipitation, the samples were washed with lysis buffer. After washing three times, the retained proteins were mixed with 30 µL of loading buffer at 100 °C for 10 min, and then protein complexes and input protein were detected by Western blotting.

### Statistical Analysis

SPSS 19 (SPSS Science Inc., Chicago, Illinois) and Prism 6.0 (Graph Pad Software, La Jolla, CA, AMERICA) were used to perform data analysis. Quantitative data are expressed as the mean ± S.E.M. A two‐tailed Student's *t*‐test was used for comparisons between control and treatment groups; and for studies involving more than two groups, data were evaluated with a one‐way analysis of variance (ANOVA) followed by the Tukey's post‐hoc‐test. For quantitative data with no homogeneity of variance or non‐parametric distribution and qualitative data, the Kruskal–Wallis H test was used prior to pairwise comparison with the Nemenyi test. Statistical significance was defined as *p* < 0.05.

## Conflict of Interest

The authors declare no conflict of interest.

## Author Contributions

H.W., Q.L., and Z.L. contributed equally to the work. H.G. and L.C. designed the research. H.G., Q.L., and Z.L. performed the research and analyzed the data. H.G. prepared the manuscript. H.G., Q.L., Y.L., K.L., X.K., Y.Z., Q.M., Z.L., X.Z., K.S., Q.X., Y.G., and L.C. revised the manuscript. All authors read and approved the final manuscript.

## Supporting information



Supporting Information

## Data Availability

The data that support the findings of this study are available from the corresponding author upon reasonable request.
