# Supplementary material for: SPP1‐ITGα5/β1 Accelerates Calcification of Nucleus Pulposus Cells by Inhibiting Mitophagy via Ubiquitin‐Dependent PINK1/PARKIN Pathway Blockade
Source: Adv Sci (Weinh). 2024 Dec 25;12(7):2411162. doi: 10.1002/advs.202411162 (PMC11831503; doi:10.1002/advs.202411162)
Supplement: Supplementary file 1 — Supporting Information [file ADVS-12-2411162-s001.docx]

**
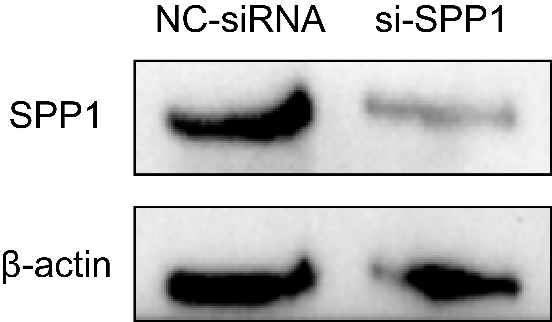
**

**Supplementary Figure S1.** The knockdown efficiency of si-SPP1**.**

**
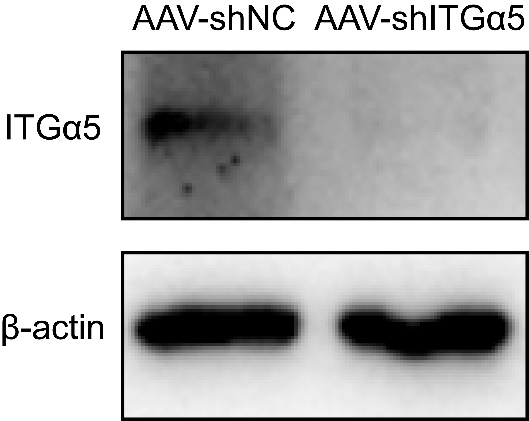
**

**Supplementary Figure S2.** The knockdown efficiency of AAV-shITGα5**.**
